# The Use of Antipsychotic Drugs for Treating Behavioral Symptoms in Alzheimer’s Disease

**DOI:** 10.3389/fphar.2019.01465

**Published:** 2019-12-06

**Authors:** Valeria Calsolaro, Rachele Antognoli, Chukwuma Okoye, Fabio Monzani

**Affiliations:** ^1^Neurology Imaging Unit, Imperial College London, London, United Kingdom; ^2^Geriatrics Unit, Department of Clinical and Experimental Medicine, University of Pisa, Pisa, Italy

**Keywords:** Alzheimer’s disease, antipsychotic drugs, D2 receptors, 5-HT2A receptors, agitation, hallucinations

## Abstract

According to the World Alzheimer’s report, dementia was estimated to affect 50 million worldwide in 2018, number expected to increase to more than 150 million within 30 years. Alzheimer’s disease is the most common type of dementia, accounting on its own for 2/3 of all dementia cases. The initial signs and symptoms of Alzheimer’s disease relate to progressive cognitive decline, inexorably progressing until the loss of independence. Neuropsychiatric and behavioral symptoms may occur during the progression of the disease; around 20% of patients without any behavioral symptoms at the diagnosis will experience some of them within 2 years. Consequences are early institutionalization, lower quality of life, of both patients and carers, and more severe cognitive impairment. Treatment options for behavioral symptoms include pharmacological and non-pharmacological approaches. The latter are usually preferred, since antipsychotic therapy is not free from several, and often serious, adverse events. However, behavioral symptoms are not always controllable with non-pharmacological intervention. The psychotropic class of medication more frequently prescribed for behavioral symptoms are atypical antipsychotics; among them, risperidone is the only one licensed for the treatment of aggression, in Europe but not in the USA. On that regard, the use of antipsychotic drugs should be limited, due to the increased risk of mortality, stroke, hallucination, and higher risk of relapse after discontinuation. Some new agents are under evaluation, such as pimavanserin and lumateperone. In this review, we are evaluating the current available pharmacological options to treat behavioral symptoms as well as the forthcoming new agents.

## Introduction

According to the World Alzheimer’s Report 2018, 50 million people are living with dementia worldwide (A.s.D. International, 2018), and Alzheimer’s disease (AD) accounts on its own for almost 2/3 of all dementia cases. AD is a devastating neurodegenerative disease characterised by progressive cognitive impairment, usually affecting, at the beginning, episodic memory. As the disease progresses, other cognitive domains become affected, leading to the loss of independence. Behavioral and psychological symptoms in dementia (BPSD) are a group of behavior, mood, perception, or thought disturbances manifesting with anxiety, agitation, delusions and hallucination (Abraha et al., 2017). The incidence of perceptual abnormalities in patient with dementia is high; around 20% of subjects with no behavioral symptoms at the diagnosis will develop some of them within 2 years (Abraha et al., 2017). Experiencing BPSD leads to institutionalization, greater cognitive impairment, worse quality of life and carers’ distress, increased mortality (Creese et al., 2018), prolonged in-hospital stay, and more difficult discharge (Davies et al., 2018). The features of the psychiatric symptoms in AD usually differ from the ones in other psychiatric disorders. Compared to schizophrenia, delusions in AD are simpler, mostly characterized by misbelief, distrust about family members, theft, and suspiciousness of abandon (De Deyn et al., 2013). Hallucinations in AD are usually visual (Lanctot et al., 2017), more rarely somatic and olfactory (El Haj et al., 2017). Agitation has been related to structural and functional impairment of emotional circuits, leading, for example, to increased perception of threat (Lanctot et al., 2017).

Unfortunately, the management of BPSD is complicated and challenging; the available licensed treatment is limited, the success of the therapy varies, and the spectrum of possible adverse events limits the choices (Lane et al., 2018). The preferred approach is the non-pharmacological one; however, despite multiple studies, there is not enough homogeneity in sample size, intervention adopted and follow-up period (Abraha et al., 2017). An evidence-based algorithm has recently been designed and proposed by multidisciplinary teams, to better manage behavioral symptoms and lead clinicians on the therapeutic choice (Davies et al., 2018).

In this review we evaluate the use of antipsychotic drugs in AD. The search was conducted in PubMed and Scopus, with keywords and search criteria as detailed in the **Supplementary Material**.

### Antipsychotic Drugs

Antipsychotic drugs can be divided in two groups: typical and atypical, depending on their strength as antagonists to dopamine D2 receptors, higher for the typical ones, and to the 5-hydroxytryptamine-A (5-HT2A) receptors, characteristic of the atypical ones (Ballard and Howard, 2006). Among the typical antipsychotic category, haloperidol remains the most prescribed; several concerns have been raised because of their low safety profile. Sedation, extrapyramidal symptoms (EPS), orthostatic hypotension, and anticholinergic effects were concerning safety issues, mainly due to the strong and long-lasting binding of D2 receptors across the whole brain regions, together with several other receptors (De Deyn et al., 2013). Risperidone, olanzapine, and quetiapine are atypical neuroleptics. The binding to D2 receptors is more targeted to selected brain regions, related to the psychotic symptoms, sparing the ones linked to the motor symptoms; these are due to an antagonist action on 5-HT2A receptors as well, or to a shorter blockage of D2 receptors (De Deyn et al., 2013). The use of antipsychotic drugs to treat agitation tracks back to the 60s (Ballard et al., 2011); since then, due to increasing evidences of safety concerns, their prescription has been widely reduced (Ballard et al., 2011).

#### Atypical Antipsychotic Drugs

Risperidone is a dopamine, serotonin, and noradrenalin receptor antagonist (Davies et al., 2018), licensed for the treatment of maniac and mixed bipolar disorder episodes and is approved for short-term treatment of behavioral symptom in dementia in UK, but not in the US (Davies et al., 2018). Several clinical trials evaluated the efficacy of risperidone in the treatment for BPSD. A significant improvement in aggression, measured with the BEHAV-AD (Behavioural Pathology in Alzheimer’s Disease) scale, was seen with risperidone at 1 and 2 mg, with better outcome on the higher dose (–1.50 [–2.05, –0.95] p < 0.0001), compared to placebo (Katz et al., 1999; Ballard and Howard, 2006). Some difference was also seen at the dose of 1 mg, compared to placebo, in treating psychosis (–0.14 [–0.25, –0.03] p = 0.01) (Katz et al., 1999; Ballard and Howard, 2006). The largest randomized controlled study (RCT) “Cognitive Effects of A typical Antipsychotic Medications in Patients with Alzheimer’s Disease” (CATIE-AD) gave interesting results. A cohort of 421 patients with AD, followed up in an outpatient setting for psychosis or agitation/aggression, was randomized to receive placebo, risperidone, quetiapine, or olanzapine for 36 weeks, with cognitive assessment at baseline, 12, 24, and 36 weeks (Vigen et al., 2011). The risperidone or olanzapine groups showed greater cognitive decline in the cognitive summary score (sum of 18 cognitive tests), while the olanzapine one had a greater decline in the mini mental state examination (MMSE); the BPRS cognitive factor was worse in quetiapine group, compared to the placebo group (Vigen et al., 2011). The phase 1 outcomes of CATIE-AD showed that, compared to placebo, the group receiving risperidone had greater improvement in the Brief Psychiatric Rating Scale (BPRS) psychosis factor and in the Clinical Global Impression of Changes (CGI-C) (Sultzer et al., 2008). The groups receiving either risperidone or olanzapine had greater improvement in the Neuropsychiatric Inventory scale (NPI) and BPRS hostile suspiciousness factor (Sultzer et al., 2008). However, the final results of the study did not show any significant advantages in the improvement in the CGI-C scale; adverse event and discontinuation due to poor tolerability favoured placebo (Schneider et al., 2006). The multicentre Antipsychotic Discontinuation in Alzheimer’s disease (ADAD) trial evaluated a cohort of 180 patients with AD and agitation or psychosis. The whole cohort received risperidone for 16 weeks, and then was randomized to proceed with risperidone for 32 weeks, receive risperidone for 16 weeks and placebo for further 16 weeks, or receive placebo for 32 weeks (Patel et al., 2017). The discontinuation of the drug led to increased risk of relapse during the randomization window, particularly for severe hallucinations at baseline (Patel et al., 2017). The dementia antipsychotic withdrawal trial (DART-AD) randomized patients with AD in care facilities to continue the current medication (thioridazine, chlorpromazine, haloperidol, trifluoperazine, or risperidone) or switch to placebo for 12 months (Ballard et al., 2009), with mortality rate as a primary outcome. Among the whole cohort, the mortality was higher in the subject continuing the medication, compared to the placebo branch (Ballard et al., 2009).

Olanzapine is an atypical antipsychotic, licensed for the treatment of schizophrenia and bipolar disorder in adults. Studies have been conducted to evaluate the efficacy in controlling behavioral symptoms in dementia. De Deyn et al. conducted a large study with > 600 AD patients with delusions or hallucinations, randomized to receive either placebo or a fixed dose of olanzapine (1.0, 2.5, 5.0, or 7.5 mg/day) for 10 weeks. An improvement in the sum of delusion and hallucination scores of the NPI was seen in the group treated, from the 2.5 mg dose, with higher efficacy for the 7.5 mg dose (De Deyn et al., 2004). Another study compared the efficacy and tolerability of a flexible dose of olanzapine and risperidone vs. placebo in the NPI and Clinical Global Impression–Severity (CGI-S) of Psychosis scale. Improvement has been noticed in all the three groups, with a higher rate of adverse events and withdrawal in the risperidone and olanzapine group (Deberdt et al., 2005). In a multicentre, double-blinded and placebo-controlled trial, 206 older patients AD patients in nursing home, showing psychotic or behavioral symptoms, were randomized to receive olanzapine (5, 10, or 15 mg/die) or placebo. Significant improvement in the summed scores of agitation/aggression, delusion and hallucination items in the NPI-nursing home version (NPI-NH) was seen in patient treated with lower doses of olanzapine, while the higher dose did not differ from the placebo. Six weeks treatment with either placebo or three different doses of olanzapine favoured the use of lower doses in controlling behavioral symptoms, compared to placebo or 15mg/day. The treatment was well tolerated. Apart from somnolence and gait disturbances, commoner in the treatment group, no other potential side effects were seen compared to placebo (Street et al., 2000).

Aripiprazole is an atypical antipsychotic drug licensed for the treatment of schizophrenia in adults and adolescents, mania in bipolar disease in children, adolescents and adults, autism, major depression in adults. Due to the safer profile, a broader use has been done, including psychosis in older patients with dementia (De Deyn et al., 2013). A 10 weeks double blind, placebo-controlled and multicentre study randomized 208 AD patients with delusions or hallucination, to receive aripiprazole 2 mg/day, possibly titrated to higher dosages, or placebo. Significant improvement was seen in the CGI-S of illness, and in the mean CGI-improvements, in patients more severely affected. Significant improvement has also been demonstrated in the BPRS psychosis and BPRS core scores, but the overall conclusion was that the improvement was only modest over placebo (De Deyn et al., 2005). A double-blind, multicentre and placebo-controlled study assigned institutionalized AD patients with psychotic symptoms to aripiprazole (2, 5 or 10 mg/day) or placebo, for 10 weeks. Significant improvement in the NPI-NH psychosis, delusion, agitation/aggression, anxiety, and irritability subscales was seen in the 10 mg treatment group. Some improvement was also seen in the Cohen-Mansfield Agitation Inventory (CMAI) scores in the 5 and 10 mg group, and the mean CGI-S was better in the 10 mg group (Mintzer et al., 2007). The higher efficacy of 10 mg of aripiprazole in controlling behavioral symptoms has been confirmed in another multicentre, placebo-controlled trial on institutionalized AD patients (Streim et al., 2008).

Quetiapine is licensed for the treatment of schizophrenia and bipolar disorder in adults, prescribed off-label to treat post-traumatic stress disorder, anxiety, insomnia, and behavioral symptoms in dementia. Two different doses of quetiapine (100 or 200 mg/daily) vs. placebo tested in a cohort of AD patients showed greater improvement, at a dose of 200 mg, in the Positive and Negative Syndrome Scale (PANSS)-Excitement Component (EC) score, as well as in the CGI-C (observation analysed as Last Observation Carried Forward (LOCF): p = 0.014 and Observed cases (OC): p = 0.002). No advantages over placebo at lower dose (Zhong et al., 2007). Quetiapine did not show any benefit compared to placebo or other antipsychotic drugs in the CATIE-AD study (Vigen et al., 2011). Another RCT compared quetiapine, rivastigmine, and placebo over 26 weeks in institutionalized AD patients; the endpoint was the improvement in agitation, measured with the CMAI scale, and cognition (Ballard et al., 2005). Neither rivastigmine nor quetiapine showed significant benefit compared to placebo, and quetiapine was associated with worse cognitive decline (Ballard et al., 2005). **Table 1** summarizes the above-mentioned studies. Overall, a meta-analysis by Ma et al. pooling RCTs with different antipsychotics, demonstrated their higher efficacy as assessed with NPI, CGI-C, CGI-S, BPRS, and CMAI, compared to placebo (Ma et al., 2014).

**Table 1:** The table summarizes the studies on atypical antipsychotic drugs included in the review

Drug	Comparison groups, study design	Dose	Outcome	AE	Ref
Risperidone	Risperidone vs. Placebo, randomized double blind, placebo controlled	1 and 2 mg 0.5 mg	BEHAV-AD scale p < 0.0001 at 2 mg BEHAV-AD psychosis sub-scale p = 0.01 at 1 mg	Dose dependent: EPS Somnolence Mild peripheral oedema for 2mg > 1mg. EPS at 1 mg NS > than placebo	(Katz et al., 1999)
	Risperidone vs. placebo/randomised controlled	Flexible dose	(-) BPRS	Reported elsewhere	(Vigen et al., 2011)
	Risperidone vs. Placebo, double blind, placebo controlled	Flexible dose	(+) BPRS psychosis factor (p = 0.01) and CGI (p < 0.001) (+) NPI total score (p = < 0.001) and BPRS suspiciousness factor (p = 0.003)	Higher withdrawn depression factor in Olanzapine group (p = 0.03)	(Sultzer et al., 2008)
	Risperidone or olanzapine vs. placebo, double blind, placebo controlled	0.5 mg or 2.5 mg	(+) NPI and CGI-S of Psychosis	Higher rate of EPS symptoms and increased prolactin levels compared to olanzapine or placebo	(Schneider et al., 2006)
Olanzapine	Olanzapine vs. placebo, double blind, placebo controlled	Flexible dose	(-) BPRS	Greater cognitive decline	(Vigen et al., 2011)
	Olanzapine vs. placebo, double blind, placebo controlled	Flexible dose	(+) NPI total score (p = 0.007) and BPRS suspiciousness factor (p = 0.006)	Higher withdrawn depression factor in Olanzapine group (p = 0.03)	(Sultzer et al., 2008)
	Olanzapine vs. placebo, double blind, placebo controlled	1mg, 2.5 mg, 5 mg or 7.5 mg	(+) NPI/NH Psychosis Total scores in all groups (p < 0.001). (+)NPI/NH Psychosis Total scores at 7.5 mg (p = 0.008) and CGI-C at 2.5 (p = 0.030).	Significant overall treatment-group differences in weight gain, Anorexia or and urinary incontinence. NS increase in EPS or total AE	(De Deyn et al., 2004)
	Olanzapine vs. placebo, multicentre double blind, placebo controlled	5mg, 10 mg or 15 mg	(+) Summed score of agitation/aggression, delusion and hallucination items in the NPI-NH with 5 or 10 mg (p < 0.001 and p = 0.006 respectively)	Higher somnolence and gait disturbances in the olanzapine group. NS difference in EPS or anticholinergic effects	(Street et al., 2000)
	Olanzapine or risperidone vs. placebo, double blind, placebo controlled	2.5 mg or 10 mg	(+) NPI CGI-S of Psychosis	Higher weight gain compared to risperidone or placebo, NS	(Schneider et al., 2006)
Aripiprazole	Aripiprazole vs. placeb, double blind, placebo controlled, multicentre	2 mg titrated according to tolerability to 5 mg, 10 mg or 15 mg	(+) in NPI psychosis subscale in treated and placebo group, NS (+)CGI-S, in patients more severely affected (p = 0.035). (+) BPRS psychosis (p = 0.029) and BPRS core scores (p = 0.042)	Higher urinary tract infections, somnolence and bronchitis. NS difference in EPS or weight gain	(De Deyn et al., 2005)
	Aripiprazole vs. placebo, double blind, placebo controlled, multicentre	2mg, 5mg or 10 mg	(+) in NPI-NH psychosis subscale (p = 0.013), CGI-S (p = 0.030), BPRS Core (0.007), CMAI (p = 0.023) and NPI-NH Psychosis response rate (p = 0.019) at 10 mg. (+) in BPRS and CMAI at 5 mg No efficacy at 2 mg	Cerebrovascular events increasing with dose.	(Mintzer et al., 2007)
	Aripiprazole vs. placebo, placebo controlled, multicentre	2 mg to be titrated to 5, 10 or 15 mg	NS in NPI-NH psychosis score (p = 0.08) or CGI-S (p = 0.19). Improvement in NPI-NH total score, BPRS, CMAI, Cornell Depression scale. (+) in CGI-S was seen in more severely affected patients	Higher somnolence in treatment group	(Streim et al., 2008)
Quetiapine	Quetiapine vs. placebo, double blind, fixed dose study	100 mg or 200 mg	(+) in PANNS-EC (OC p = 0.014) and CGI-C (OC p = 0.002) at 200 mg. No difference with placebo at 100 mg	Mortality numerically higher in the quetiapine group	(Zhong et al., 2007)
	Quetiapine vs. placebo, double blind, placebo controlled	Flexible dose	No benefit	Greater cognitive decline	(Vigen et al., 2011)
	Quetiapine vs. placebo, randomised, placebo controlled		No improvement in agitation inventory scores	Significantly greater cognitive decline	(Ballard et al., 2005)

BEHAV-AD, Behavioural Pathology in Alzheimer’s Disease; BPRS, Brief Psychiatric Rating Scale; NPI, Neuropsychiatric Inventory; NPI/NH, nursing home version; CGI-S, Clinical Global Impression–Severity; CGI-C, Clinical Global Impression of Changes; CMAI, Cohen-Mansfield Agitation Inventor; PANNS-EC, Positive and Negative Syndrome Scale (PANSS)-Excitement Component (EC).

### Antipsychotics and Safety

Neuroleptic drugs have been related to several adverse event and safety issues. The increasing concerns about adverse events and tolerability led the FDA to include a warning, in the form of a “black box” in the atypical and typical antipsychotic labels, to limit the prescription (Dorsey et al., 2010). The warning has also been issued by the European Medicines Agency (EMA); the committee for Medicinal Products for Human Use recommended including a warning of increased risk of death for all conventional antipsychotics in the older patients with dementia. Among the most frequent and widely reported side effect of neuroleptic, drowsiness, tardive dyskinesia and parkinsonism have been reported (Ballard and Howard, 2006). Moreover, increased risk of venous thromboembolic events (VTE) has been demonstrated in patients treated with antipsychotics, both in general and older population (Jonsson et al., 2018). In a meta-analysis in 2006, Ballard *et al*. reported several side effects of neuroleptics; haloperidol, among the first generation ones, was associated with EPS and drowsiness (Ballard and Howard, 2006). A systematic review aiming to compare the rates of EPS with second generation antipsychotic drugs with the first-generation ones, confirmed a reduced incidence with the former, across different pathologies and age groups (Correll et al., 2004). Among atypical neuroleptics, risperidone showed increased EPS at the dose of 1 mg and 2 mg; dose-dependent increase in somnolence and peripheral oedema was also seen in institutionalized AD patients (Katz et al., 1999). The higher incidence of EPS, somnolence, urinary tract infection (UTI), has been confirmed in two more recent studies, among nursing home residents with AD, vascular dementia or mixed (Mintzer et al., 2006). Gait disturbances and somnolence were also more common in patients treated with olanzapine compared to placebo (Street et al., 2000). Moreover, the impact on cardiovascular system is also a concern. In particular, elongation of the QT interval, torsade de pointes (TdP), and sudden cardiac death have a known relationship with antipsychotic drugs (Sicouri and Antzelevitch, 2018). A case control study on a population aged 65 years or above, undertaking antipsychotics at possible or conditional risk of TdP, demonstrated higher TdP risk for drugs classified as “known TdP risk, ” with risk ranking of haloperidol > risperidone > olanzapine > quetiapine (Danielsson et al., 2016). The risk of VTE is estimated to be double, compared to the general population, especially within the first three months of therapy, and several mechanisms are potentially responsible for this association. The reduced physical activity of older patients seems to play a role and, risk increases when associating more than one neuroleptic (Oglodek et al., 2018). Patients receiving typical antipsychotics have been reported to have elevated levels of antiphospholipid antibodies (including anticoagulants and anticardiolipin antibodies), which are associated with an increased VTE risk. Moreover, atypical antipsychotics may cause metabolic syndrome, which is per se a VTE risk factor (Oglodek et al., 2018).

Increased incidence of cerebrovascular disease has been demonstrated in randomized controlled studies in older patients (Herrmann and Lanctot, 2005), although the matter is debated. A recent meta-analysis demonstrated a more than doubled risk of stroke in general population using antipsychotics, which although appears to be lower in population with dementia. No association was seen with myocardial infarction, although the heterogeneity of the available studies limits the strength of the conclusion (Zivkovic et al., 2019). The meta-analysis by Ma et al., together with the efficacy of the antipsychotic therapy, showed higher risk for adverse events, such EPS (OR 1.74), cerebrovascular events (OR 2.5), somnolence (OR 2.95), gait disturbances (OR 3.35), oedema (OR 1.8), UTI (OR 1.35), and death (OR 1.52) (Ma et al., 2014). A large meta-analysis was published in 2018, including more than 380,000 patients with dementia, including more than 80,000 using antipsychotics, and 359,235 patients without dementia (Ralph and Espinet, 2018). The results showed the HR (hazard ratio) for all-cause mortality in patients treated with antipsychotic drugs 1.9–2.19, with higher risk within the first 180 days, and dose related. The risk was similar in subjects with and without dementia (Ralph and Espinet, 2018).

Treating patients with dementia often means treating older patients, presenting with polypathology, polypharmacy, and different sensitivity to the effect of psychotropic drugs. For this reason, potential drug drug-drug interaction needs to be considered (Pasqualetti et al., 2015). Taken together, the need of new therapeutic options, with a safer profile, is urgently needed, to better address such a delicate problem like the management of older patients and often frail patients.

### Different Approaches and New Options

Different pharmacological approaches for the treatment of behavioral symptoms have been evaluated.

Acetylcholinesterase inhibitors (AChEI) demonstrated a mild effect on BPSD, particularly on agitation, delusion, aggression, or hallucination (Masopust et al., 2018); more benefits have been seen in Lewy Body dementia-related hallucinations (Creese et al., 2018). A recent meta-analysis demonstrated the efficacy of memantine in controlling “positive” symptoms such as aggression, agitation, delusions, disinhibition, compared to control; it was also effective on hallucinations (Kishi et al., 2017). The combined use of AChEI+memantine has been evaluated in a meta-analysis by Matsunaga et al; the results showed a better outcome of the combined therapy compared to AChEI alone in both behavioral and functional scores, with also a positive trend in cognitive performance (Matsunaga et al., 2014). A further meta-analysis confirmed the better outcome in terms of both cognitive and behavioral symptoms of the combined therapy (Chen et al., 2017).

Among selective serotonin uptake inhibitors, sertraline showed some efficacy in a cohort of patients with mild to moderate agitation; citalopram gave good results on agitation in the face, however, of possible QTc prolongation. (Davies et al., 2018). When compared to atypical antipsychotic, such as perphenazine, risperidone, or quetiapine, improvement in agitation has been seen, with less adverse event for the citalopram-treated group (Ahmed et al., 2019). Trazodone has been tested as well; reduction in agitation could be a consequence of sedation due to its histaminergic effect (Davies et al., 2018). Anticonvulsants, such as carbamazepine and gabapentin, showed some potential effects, however in case reports or small and not RCT trials (Creese et al., 2018; Davies et al., 2018).

An algorithm for the therapeutic approach to agitation and aggression in patients with AD or mixed dementia, revised by a multidisciplinary team, was recently proposed (Davies et al., 2018). The algorithm hypothesizes a sequential use of medication, basing on evidences, efficacy, safety and tolerability. Risperidone is the first step of treatment (Davies et al., 2018), followed by quetiapine or aripiprazole; titration and switching-drug time-point are also suggested. (Davies et al., 2018).

Atypical antipsychotic drugs, such as primavanserin, lumateperone, and brexpiprazole, are currently under evaluation. Primavanserin, approved for the treatment of hallucinations and delusions in Parkinson’s disease, works reducing the baseline activity of 5HT2A receptors, which are upregulated in psychotic symptoms, mediating the effects of 5HT2C without antagonizing D2 receptors. A phase II, double blind, placebo-controlled study in UK showed significant improvement in the NPI-NH psychosis score in the treatment group compared to placebo, at 6 weeks of treatment (Ahmed et al., 2019). However, concerns were raised about the reliability of the results, due to the small effect, only observed at 6 weeks, and the potential safety issues (Schneider, 2018). Nonetheless, another double blind, placebo-controlled study is undergoing to evaluate its efficacy in preventing relapse of psychotic symptoms (Ahmed et al., 2019). A phase III double blind, placebo-controlled study in AD patients with clinically significant agitation, is currently undergoing with lumateperone, a first-in-class agent in development for schizophrenia that acts synergistically through serotonergic, dopaminergic and glutamatergic systems (Porsteinsson and Antonsdottir, 2017). Brexpiprazole, a novel serotonin-dopamine receptor modulator with partial agonist activity at serotonin_1A_ and dopamine_2/3_ receptors, has been approved for the treatment of schizophrenia and major depressive disorders (Porsteinsson and Antonsdottir, 2017). Two Phase III, randomized, double blind, placebo-controlled studies were conducted in AD patients with agitation; only one showed improvement in the CMAI scores. However, a further Phase III study is ongoing (Ahmed et al., 2019).

## Conclusions

AD is a growing healthcare, social, and economic problem. The increased life expectancy with the consequent progressive population ageing lead to higher incidence and prevalence of age-related, chronic diseases, mirrored by the complexity of older patients. Despite the improved diagnostic approach to AD, no disease-modifying medications exist. Behavioral symptoms represent part of the complexity of dementia, and are related to worse cognitive outcome; their treatment is particularly challenging, especially when facing complex and frail patients. Nonetheless, non-pharmacological treatments have shown promising effects in reducing behavioral and psychological symptoms of dementia. Among the available treatments licensed for psychiatric diseases, only risperidone is approved in Europe for behavioral disorders in dementia; other antipsychotics are prescribed “off label.” However, some trials are currently undergoing with new potential medications with safer profile.

## Author Contributions

VC, RA, and CO equally contributed to scientific literature research and analysis. VC wrote the manuscript. FM designed the study, analyzed the scientific literature, and revised the manuscript.

## Conflict of Interest

The authors declare that the research was conducted in the absence of any commercial or financial relationships that could be construed as a potential conflict of interest.
