# Supplementary material for: The Use of Antipsychotic Drugs for Treating Behavioral Symptoms in Alzheimer’s Disease
Source: Front Pharmacol. 2019 Dec 6;10:1465. doi: 10.3389/fphar.2019.01465 (PMC6915160; doi:10.3389/fphar.2019.01465)
Supplement: Supplementary file 1 [file Table_1.docx]

**Literature search criteria**

The literature search covered papers published in PubMed and Scopus, using the keywords “antipsychotics and dementia”, “antipsychotic Alzheimer’s disease”, “risperidone in dementia”, “quetiapine in dementia” “olanzapine in dementia”, “aripiprazole in dementia”, antipsychotic safety in dementia”, “agitation/hallucination/psychosis in dementia”. We only selected papers in English, studies on geriatric population and randomised clinical trials, either to placebo or to different therapeutic approach. We included only studies with not less than 90 subjects enrolled, published within the last 20 years. We have also searched recent meta-analysis and systematic reviews. We excluded literature not in English, case reports or studies not in geriatric population, apart from papers explaining possible pathologic mechanisms*.*
